# The contribution of commonly consumed edible insects to nutrition security in the Eastern D.R. Congo

**DOI:** 10.1038/s41598-024-64078-5

**Published:** 2024-07-13

**Authors:** Jackson Ishara, Rehema Matendo, Jeremiah Ng’ang’a, Shahida Anusha Siddiqui, Saliou Niassy, Karume Katcho, John Kinyuru

**Affiliations:** 1 Department of Food Science and Technology, Université Evangélique en Afrique, P.O. Box 3323, Bukavu, Democratic Republic of the Congo; 2 Department of Food Science and Technology, Jomo Kenyatta University of Agriculture and Technology, P.O. Box 62000-00200, Nairobi, Kenya; 3 Faculty of Agriculture and Environmental Sciences, Université de Kaziba, P.O. Box 2106, Bukavu, Democratic Republic of the Congo; 4 Department of Environmental and Agronomic Sciences, Université Officielle de Bukavu, P.O. Box 570, Bukavu, Democratic Republic of the Congo; 5 German Institute of Food Technologies (DIL E.V.), Prof.-von-Klitzing Str. 7, D-49610 Quakenbrück, Germany; 6 Inter-African Phytosanitary Council of African Union (AU-IAPSC), P.O Box 4170, Yaoundé, Cameroon; 7 Department of Zoology and Entomology, Faculty of Natural and Agricultural Sciences, University of Pretoria, Hatfield, Pretoria, Gauteng South Africa; 8 Faculty of Agriculture and Environmental Sciences, Université Evangélique en Afrique, Bukavu, 3323 Democratic Republic of the Congo; 9 Centre de Recherche en Géothermie, Bukavu, 327 Democratic Republic of the Congo; 10 African Institute for Capacity Development (AICAD), P.O. Box 46179-00100, Nairobi, Kenya

**Keywords:** Edible insects, Nutritional potential, Sensory attributes, Food insecurity, Biochemistry, Biotechnology

## Abstract

Edible insects are perceived as an incredible opportunity to mitigate the major challenge of sustainably producing healthy foods for a growing world population in the face of climate change uncertainties over the coming decade. In this study, we assessed the nutrient composition and sensory properties of *Acheta domesticus*, *Apis mellifera*, *Gnathocera trivittata*, *Gryllotalpa africana*, *Imbrasia epimethea*, *Imbrasia oyemensis*, *Locusta migratoria*, *Macrotermes subhylanus*, *Nomadacris septemfasciata*, *Rhyncophorus phoenicis*, *Ruspolia differens* and *Rhynchophorus ferrugineus* consumed in Eastern D. R. Congo. The investigated edible insects are highly appreciated and nutritious, with proteins (20.67–43.93 g/100 g) and fats (14.53–36.02 g/100 g) being the major macro-nutrients, proving their potential to improve diets through food enrichment. The high potassium (24–386.67 mg/100 g), sodium (152–257.82 mg/100 g), magnesium (32–64 mg/100 g), iron (5.3–16.13 mg/100 g), calcium (25–156.67 mg/100 g) and zinc (11–19.67 mg/100 g) content make the assessed edible insects a useful mineral-containing ingredient for preventing undernutrition in countries which are plagued by micronutrient deficiencies. A scatter plot of matrices and Pearson’s correlations between sensory attributes and nutritional composition showed a negative correlation (r = − 0.45) between protein and appearance. While no strong correlation was observed between nutritional attributes and sensory acceptance, a positive correlation was observed between potassium and aroma (r = 0.50), after-taste (r = 0.50) and acceptability (r = 0.52). Principal component analysis results indicated that the two axes accounted for up to 97.4% of the observed variability in the nutrient composition and sensory attributes of commonly consumed edible insects in the Eastern D. R. Congo. Given the significant delicacy and nutritional potential of edible insects highlighted in this paper, households can rely on the latter to meet their nutritional needs rather than conventional livestock, thus contributing to environmental and financial security through local business opportunities.

## Introduction

In the face of growing world population^1^, climate change uncertainties^2,3^, economic pressures and increasingly demanding consumers^4^, edible insects offer an opportunity to produce sustainably and sufficiently to tackle the serious challenge of feeding people in the coming decades^5–7^. Given their nutritional potential^8^, taste^9,10^, economic benefits^11,12^, less land-dependent production^13^, high feed conversion efficiency compared to conventional livestock and relatively low emission of greenhouse gases^14^, and ammonia^15^, with sufficient biomass in some wild-harvested for commercial supply^16^, intensive rearing at both household level^17^, and industrial scale is recommended^18^.

Furthermore, edible insects are consumed by around 2 billion people^19^, mainly in parts of Asia, Africa and Latin America, with over 2100 species already listed as edible^20^, in over 110 countries worldwide^21^. Thus, edible insects can offset the increasing demand for animal protein and avoid deforestation for pasture use^22^. Among the most consumed insect groups, we find a diversity of beetles (Coleoptera, 31%), caterpillars (Lepidoptera, 18%), bees, wasps and ants (Hymenoptera, 14%), locusts, grasshoppers, crickets and crickets (Orthoptera, 13%), cicadas, leafhoppers, grasshoppers, mealybugs and bedbugs (Hemiptera, 10%), termites (Isoptera, 3%), dragonflies (Odonata, 3%), flies (Diptera, 2%) and 5% other orders^23^.

The Republic Democratic of Congo (DRC) is known for its wide biodiversity including edible insects^24–27^, especially as the collection and trade of the latter and other non-timber forest products are legal activities encouraged by the 2002 DRC Forestry Code adopted by Law No. 11/2002 to encourage sustainable management and socio-economic benefits for local communities^28^. However, there is a lack of specific regulations addressing the unique challenges and opportunities associated with native foods in the country despite the existence of broader policies related to biodiversity management, land use, forestry regulations and agriculture^29^. It is therefore imperative that policies, legislation and regulations to promote the latter be prioritized to encourage their production, marketing and consumption.

Often, research on insect consumption focuses on protein content, whereas high levels of important micronutrients in insects, particularly iron and zinc, can be of significant importance^30^ as much as the one reported in nutritious foods such as mushrooms^31^. This is particularly critical, as micronutrient deficiencies are widespread in developing countries, especially among children and breastfeeding women^32^. Edible insects have excellent protein quality^33^, with good amino acid content, energy content, fatty acid profiles and high levels of various micronutrients such as magnesium, manganese, phosphorus, selenium and zinc, as well as the vitamins riboflavin, pantothenic acid, biotin and, in some cases, folic acid^34^. Edible insects also meet the principles of sustainability, accessibility and palatability^35^.

Unlike traditional livestock, standardized information on commercially available edible insects' nutritional composition and sensory quality is limited and inconclusive^36^. However, these limited data are increasingly used to justify generalized claims about the health benefits of a particular genus, order or even insects as a homogeneous food category^23,37^. In several countries, efforts are being made in this direction. However, in the DRC, several species are not yet nutritionally characterized, thus posing a concern about strategies to develop rich foods that would help mitigate food shocks. Given the relevance of edible insects in contributing to food security, it is imperative to assess their true potential. Thus, this study focused not only on assessing the nutritional potential of edible insects in Eastern D.R. Congo, where anthropo-entomophagy practices are widespread, but also on establishing a valuable baseline that could help develop nutritious diets for vulnerable populations still suffering from food insecurity and malnutrition.

## Results

### Macronutrient composition of commonly consumed edible insects

The macronutrient composition of the edible insects varied significantly (*p* < 0.05) except for ash content, as depicted in Table 1. While protein content ranged between 20 and 43 g/100 g, fat content ranged between 14 and 36 g/100 g, ash (4 and 6 g/100 g), and moisture content (59 and 77 g/100 g). Concerning protein content, *I. oyemensis* had the highest protein content, followed by *I. epimethea*, *A. domesticus*, *R. ferrugineus*, *R. phoenicis*, *G. trivittata*, *R. differens*, *G. africana*, *L. migratoria*, *N. septemfasciata*, *M. subhyalinus*, and *A. mellifera* had the lowest protein content. As for fat content, species such as *R. differens*, *R. phoenicis*, *M. subhyalinus*, *N. septemfasciata* and *A. mellifera* had high fat content, and species such as *G. africana*, *A. domesticus*, *I. oyemensis*, *R. ferrugineus*, *L. migratoria*, *G. trivittata* and *I. epimethea* had the lowest fat content in comparison to the latter. As for moisture content, *I. epimethea* has the highest moisture content, followed by *A. mellifera*, *I. oyemensis*, *G. africana*, *L. migratoria*, *R. phoenicis*, *R. differens*, *R. ferrugineus*, *M. subhyalinus*, *A. domesticus*, *N. septemfasciata* and *G. trivittata*. Studied edible insects were found to have no significant differences (*p* > 0.05) in their ash content, which ranged between 4.86 and 6.97 g/100 g, with *R. phoenicis* having the highest, followed by *A. domesticus*, *I. oyemensis*, *M. subhyalinus*, *R. ferrugineus*, *I. epimethea*, *G. trivittata*, *A. mellifera*, *R. differens*, *L. migratoria*, *N. septemfasciata*, and *G. africana* had the lowest.

**Table 1:** Macronutrient composition of commonly consumed edible insects.

Insect species	MC (g/100 g)	Protein (g/100 g)	Fat (g/100 g)	Ash (g/100 g)
*Acheta domesticus*	61.68 ± 2.00de	37.05 ± 1.49b	22.28 ± 1.85bcde	6.69 ± 0.54a
*Apis mellifera*	72.93 ± 2.01ab	20.67 ± 1.43e	24.36 ± 1.84bc	5.70 ± 0.53a
*Gnathocera trivittata*	59.31 ± 2.83e	35.26 ± 2.11bc	17.34 ± 2.61de	5.75 ± 0.76a
*Grillotalpa africana*	71.69 ± 2.80ab	31.46 ± 2.01 cd	23.98 ± 2.60bcd	4.86 ± 0.76a
*Imbrasia epimethea*	77.54 ± 4.01a	39.04 ± 2.99ab	14.53 ± 3.67e	5.76 ± 1.08a
*Imbrasia oyemensis*	72.82 ± 2.83ab	43.93 ± 2.11a	21.81 ± 2.62bcde	6.43 ± 0.76a
*Locusta migratoria*	68.85 ± 2.00abc	31.31 ± 1.49 cd	20.79 ± 1.84cde	5.23 ± 0.54a
*Macrotermes subhyalinus*	64.36 ± 2.31cde	27.55 ± 1.72d	26.10 ± 2.13bc	6.16 ± 0.62a
*Nomadacris septemfasciata*	61.37 ± 2.83de	27.57 ± 2.12d	25.12 ± 2.60bc	4.90 ± 0.76a
*Rhynchophorus phoenicis*	68.49 ± 2.82abcd	35.37 ± 2.10bc	28.20 ± 2.59ab	6.97 ± 0.76a
*Ruspolia differens*	66.96 ± 4.00abcde	34.47 ± 2.98bc	36.02 ± 3.70a	5.56 ± 1.08a
*Rynchophorus ferrugineus*	66.00 ± 4.01bcde	35.75 ± 2.89bc	21.49 ± 3.69bcde	5.80 ± 1.07a
*p*-value	< 0.001	< 0.001	0.004	0.206

Mean values (n = 3) ± SE. All values except moisture are expressed on dry weight.

Values in the same column with the same following letter do not significantly differ (*p* < 0.05).

MC: Moisture content.

### Mineral profile

The mineral composition of commonly consumed edible insects in the Eastern of D. R. Congo, namely *A. domesticus*, *A. mellifera*, *G. trivittata*, *G. africana*, *I. epimethea*, *I. oyemensis*, *L. migratoria*, *M. subhyalinus*, *N. septemfasciata*, *R. phoenicis*, *R. differens* and *R. ferrugineus* is presented in Table 2. Potassium, sodium, magnesium, iron, calcium and zinc content varied significantly (*p* < 0.05) among edible insect species. Generally, potassium content ranged between 24 mg/100 g and 386.67 mg/100 g, sodium (152.27–257.82 mg/100 g), magnesium (32-64 mg/100 g), iron (5.30–16.13 mg/100 g), calcium (25.67–156.67 mg/100 g) and zinc (11–19.67 mg/100 g). Concerning potassium content, *M. subhyalinus* had the highest content, followed by *R. differens*, *I. epimethea*, *I. oyemensis*, *A. domesticus*, *A. mellifera*, *L. migratoria*, *N. septemfasciata*, *G. africana*, *G. trivittata*, *R. phoenicis* and *R. ferrugineus* had the lowest potassium content. Sodium content was highest in *R. differens*, followed by *R. phoenicis*, *R. ferrugineus*, *N. septemfasciata*, *M. subhyalinus*, *L. migratoria*, *I. epimethea*, *G. africana*, *A. domesticus*, *G. trivittata* and *A. mellifera* had the lowest sodium content.

**Table 2:** Mineral composition of commonly consumed edible insects in Eastern D. R. Congo (mg/100 g).

Insect species	Potassium	Sodium	Magnesium	Iron	Calcium	Zinc
*Acheta domesticus*	117.75 ± 18.55	156.33 ± 1.72 cd	48.17 ± 3.53bc	5.71 ± 0.32e	126.17 ± 5.28 cd	14.08 ± 0.49 cd
*Apis mellifera*	91.83 ± 18.50de	152.27 ± 1.73f.	48.83 ± 3.54abc	6.80 ± 0.30 cd	130.42 ± 5.23bc	14.16 ± 0.44 cd
*Gnathocera trivittata*	61.83 ± 26.23de	152.77 ± 2.44ef	43.00 ± 4.99bcd	7.65 ± 0.45b	138.33 ± 7.47abc	14.33 ± 0.70 cd
*Grillotalpa africana*	63.50 ± 26.06de	154.73 ± 2.49e	32.83 ± 4.89d	5.31 ± 0.44f.	145.67 ± 7.44ab	15.20 ± 0.69bc
*Imbrasia epimethea*	191.00 ± 37.09bc	156.70 ± 3.45 cd	64.00 ± 7.06a	6.44 ± 0.64 cd	106.00 ± 10.56def	19.67 ± 0.99a
*Imbrasia oyemensis*	124.17 ± 26.22 cd	156.84 ± 2.44 cd	63.17 ± 4.90a	7.52 ± 0.45b	104.67 ± 7.47ef	13.00 ± 0.70de
*Locusta migratoria*	86.16 ± 18.54de	157.90 ± 1.73 cd	53.50 ± 3.53ab	5.81 ± 0.32e	133.08 ± 5.28bc	13.75 ± 0.49 cd
*Macrotermes subhyalinus*	386.67 ± 21.41a	161.97 ± 1.99c	38.67 ± 4.08 cd	5.56 ± 0.37ef	123.89 ± 6.10cde	16.33 ± 0.57b
*Nomadacris septemfasciata*	85.00 ± 26.23de	162.38 ± 2.44	42.67 ± 4.99bcd	7.29 ± 0.45b	156.67 ± 7.47a	12.67 ± 0.69de
*Rhynchophorus phoenicis*	27.83 ± 26.00e	170.00 ± 2.40b	45.33 ± 4.90bcd	7.00 ± 0.44bc	137.17 ± 7.44abc	15.17 ± 0.70bc
*Ruspolia differens*	272.67 ± 37.09b	257.82 ± 3.45a	32.33 ± 7.06d	16.13 ± 0.64a	25.67 ± 10.56 g	13.33 ± 0.99cde
*Rynchophorus ferrugineus*	24.00 ± 37.00e	169.10 ± 3.44bc	32.00 ± 7.04d	5.30 ± 0.63f.	97.00 ± 10.55f.	11.00 ± 0.98e
*p*-value	< 0.001	< 0.001	< 0.001	< 0.001	< 0.001	< 0.001

Mean values (n = 3) ± SE. Values in the same column with the same following letter do not significantly differ (*p* < 0.05).

As for magnesium, the highest content was found in *I. epimethea*, followed by *I. oyemensis*, *L. migratoria*, *A. mellifera*, *A. domesticus*, *R. phoenicis*, *G. trivittata*, *N. septemfasciata*, *M. subhyalinus*, *G. africana*, *R. differens* and *R. ferrugineus* had the lowest. Commonly edible insects in the eastern part of D. R. Congo are rich in iron and zinc. *Ruspolia differens* was found to have the highest iron content, followed by *G. trivittata*, *N. septemfasciata*, *R. phoenicis*, *A. mellifera*, *I. epimethea*, *L. migratoria*, *A. domesticus*, *M. subhyalinus*, *G. fricana* and *R. ferrugineus* had the lowest. In regard to zinc content, *I. epimethea* had the highest followed by *M. subhyalinus*, *G. africana*, *R. phoenicis*, *G. trivittata*, *A. mellifera*, *A. domesticus*, *L. migratoria*, *R. differens*, *I. oyemensis*, *N. septemfasciata* and lowest zinc content was found in *R. ferrugineus*.

### Sensory acceptance

The sensory acceptance of commonly consumed edible insects is presented in Table 3 and varied significantly (*p* < 0.05) among edible insect species. Based on overall acceptability, *M. subhyalinus* had the highest sensory score edible insect, followed by *R. differens*, *N. septemfasciata*, *R. phoenicis*, *L. migratoria*, *G. africana*, *I. epimethea*, *A. mellifera*, *R. ferrugineus*, *I. oyemensis*, *G. trivittata* and *A. domesticus* had the lowest sensory scores. As for taste, *M. subhyalinus* and *R. differens* were the most appreciated species, followed by *R. phoenicis*, *N. septemfasciata*, *L. migratoria*, *I. epimethea*, *G. africana*, *A. mellifera*, *I. oyemensis*, *G. trivittata*, *R. ferrugineus* and *A. domesticus* had the lowest taste score.

**Table 3:** Sensory acceptance of commonly consumed edible insects in Eastern D. R. Congo.

Insect species	Appearance	Aroma	Texture	Taste	After taste	Overall acceptability
*Acheta domesticus*	3.76 ± 0.10d	4.39 ± 0.09f.	4.01 ± 0.08d	3.91 ± 0.09 h	3.81 ± 0.09 h	3.61 ± 0.05i
*Apis mellifera*	4.77 ± 0.09c	4.55 ± 0.08e	4.00 ± 0.09d	4.64 ± 0.08ef	4.49 ± 0.08ef	4.03 ± 0.05 fg
*Gnathocera trivittata*	3.35 ± 0.13e	4.77 ± 0.13 cd	3.89 ± 0.12d	4.29 ± 0.13 g	4.16 ± 0.12gh	3.70 ± 0.08hi
*Grillotalpa africana*	3.80 ± 0.14d	4.82 ± 0.12 cd	4.96 ± 0.11b	4.99 ± 0.12de	4.69 ± 0.12de	4.31 ± 0.07e
*Imbrasia epimethea*	3.87 ± 0.19d	3.84 ± 0.18 h	4.98 ± 0.17b	5.03 ± 0.18cde	4.68 ± 0.18de	4.22 ± 0.11ef
*Imbrasia oyemensis*	3.83 ± 0.14d	4.12 ± 0.12 fg	4.43 ± 0.12c	4.56 ± 0.12 fg	4.41 ± 0.12efg	3.89 ± 0.08gh
*Locusta migratoria*	4.89 ± 0.10bc	5.50 ± 0.08c	5.04 ± 0.08b	5.26 ± 0.09 cd	5.02 ± 0.09 cd	4.70 ± 0.05d
*Macrotermes subhyalinus*	5.34 ± 0.11a	6.24 ± 0.10a	5.48 ± 0.10a	6.23 ± 0.10a	6.27 ± 0.10a	5.55 ± 0.06a
*Nomadacris septemfasciata*	4.68 ± 0.14c	5.62 ± 0.12bc	5.63 ± 0.12a	5.44 ± 0.13bc	5.11 ± 0.12bc	4.91 ± 0.08bc
*Rhynchophorus phoenicis*	5.16 ± 0.13ab	5.01 ± 0.13c	4.99 ± 0.12b	5.63 ± 0.12b	5.22 ± 0.11bc	4.80 ± 0.07 cd
*Ruspolia differens*	5.48 ± 0.19a	5.91 ± 0.18b	5.35 ± 0.17ab	6.15 ± 0.18a	5.41 ± 0.18b	5.10 ± 0.11b
*Rynchophorus ferrugineus*	4.10 ± 0.19d	4.56 ± 0.17e	4.23 ± 0.16c	4.15 ± 0.18gh	4.18 ± 0.18fgh	3.90 ± 0.11gh
*p*-value	< 0.001	< 0.001	< 0.001	< 0.001	< 0.001	< 0.001

Mean values (n = 40) ± SE. Values in the same column with the same following letter do not significantly differ (*p* < 0.05).

Based on appearance, *R. differens* had the highest sensory score, followed by *M. subhyalinus*, *R. phoenicis*, *L. migratoria*, *A. mellifera*, *N. septemfasciata*, *R. ferrugineus*, *I. epimethea*, *I. oyemensis*, *G. africana*, *A. domesticus* and *G. trivittata* had the least appreciated appearance. Regarding aroma, *M. subhyalinus* had the highest aroma score, followed by *R. differens*, *N. septemfasciata*, *L. migratoria*, *R. phoenicis*, *G. africana*, *G. trivittata*, *R. ferrugineus*, *A. mellifera*, *A. domesticus*, *I. oyemensis* and *I. Epimethea* had the lowest aroma score.

As for texture and aftertaste, *N septemfasciata* and *M. subhyalinus* were the most appreciated. After *N septemfasciata, Macrotermes subhyalinus*, *R. differens* and *L. migratoria* were the most appreciated for texture. *Gnathocera trivittata*, *A. mellifera*, *A. domesticus* and *R. ferrugineus* had the lowest score for texture. Similarly, *R. differens*, *R. phoenicis*, *N. septemfasciata* and *L. migratoria* were the most appreciated for aftertaste just after *M. subhyalinus*. *Acheta domesticus*, *G. trivittata*, *R. ferrugineus* and *I. oyemensis* were the least appreciated for their aftertaste.

**Figure 1:**
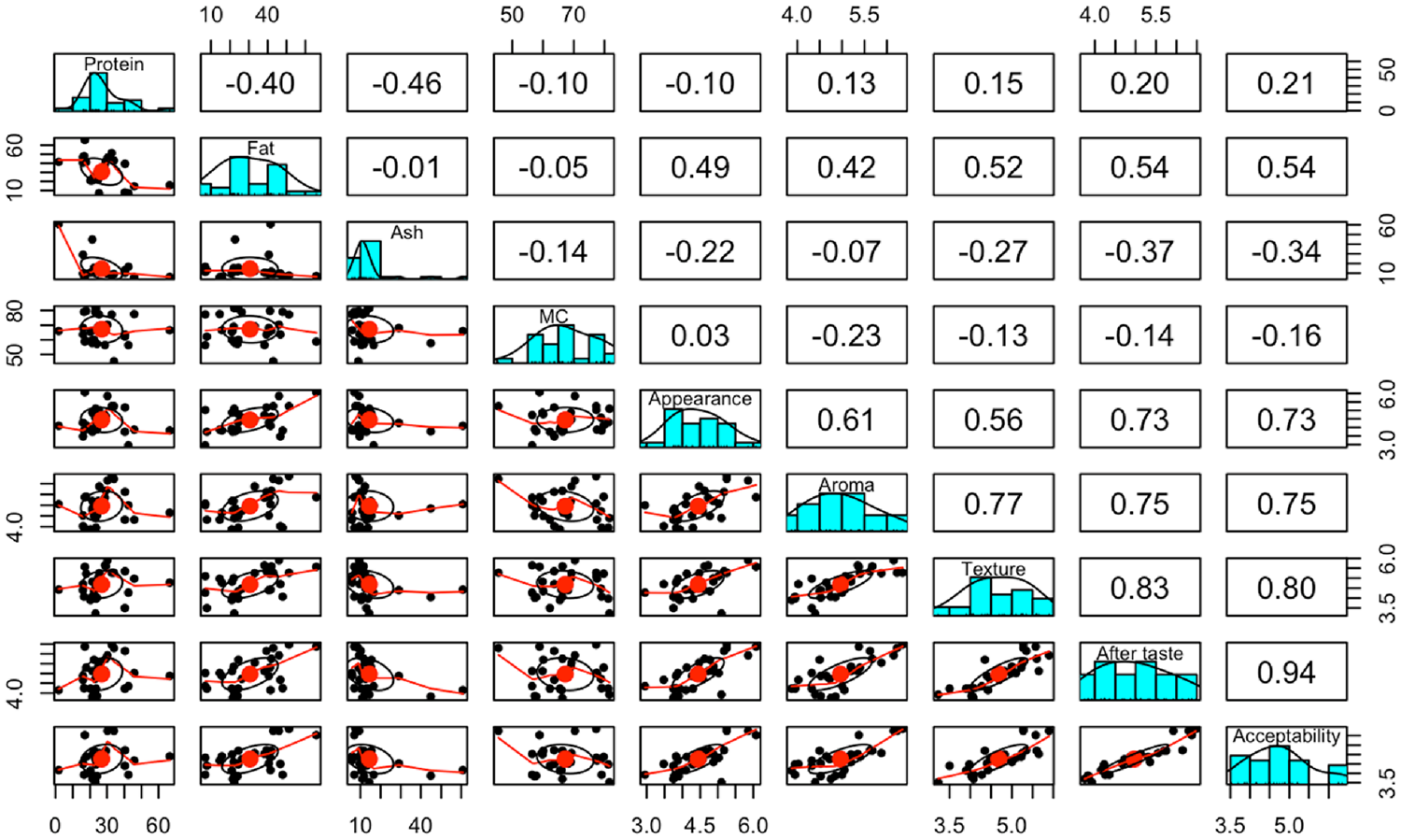
Scatter plot of matrices (SPLOM), histograms, and Pearson correlations between sensory scores and nutrient composition.

**Figure 2:**
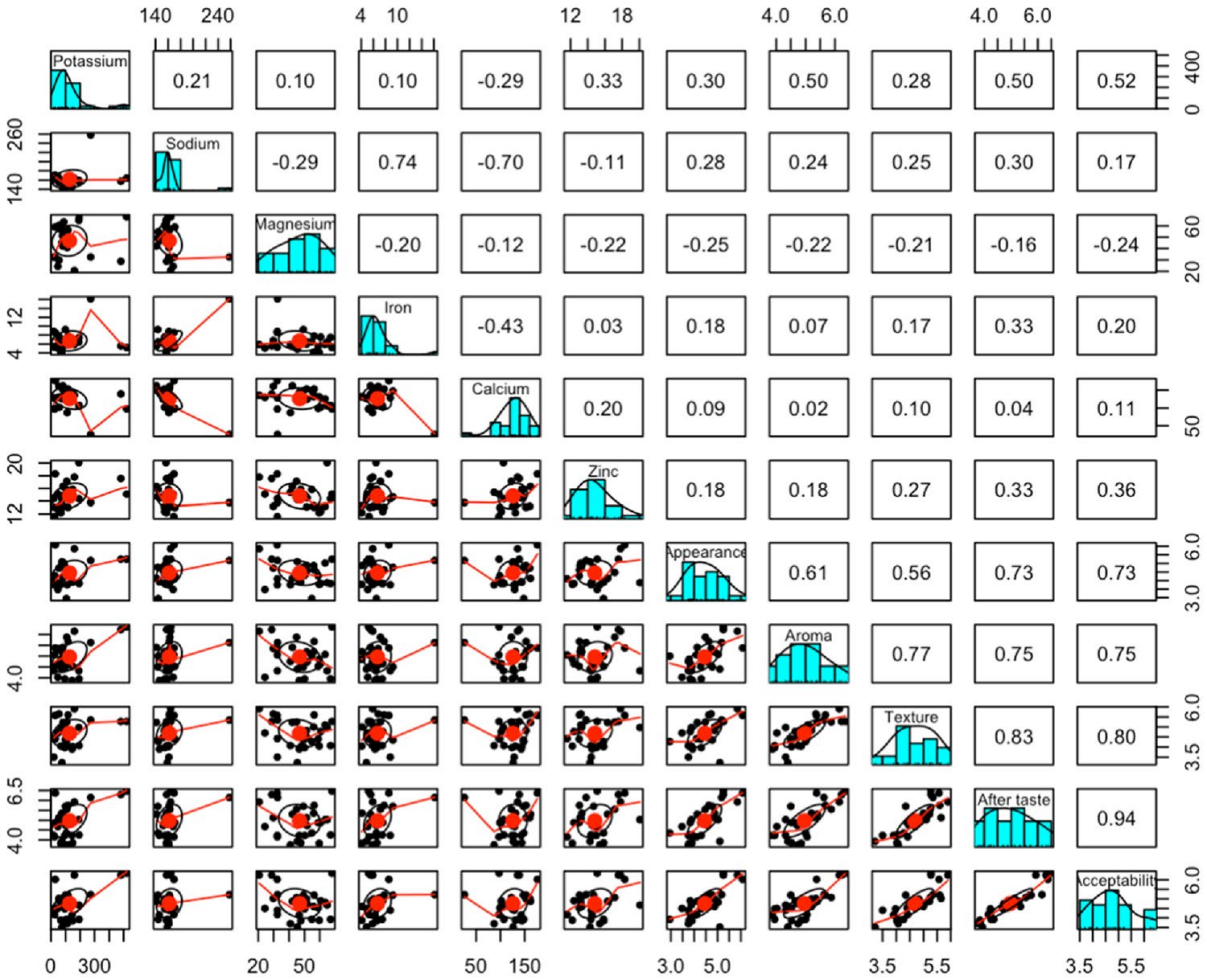
Scatter plot of matrices (SPLOM), histograms, and Pearson correlations between sensory and mineral content.

**Figure 3:**
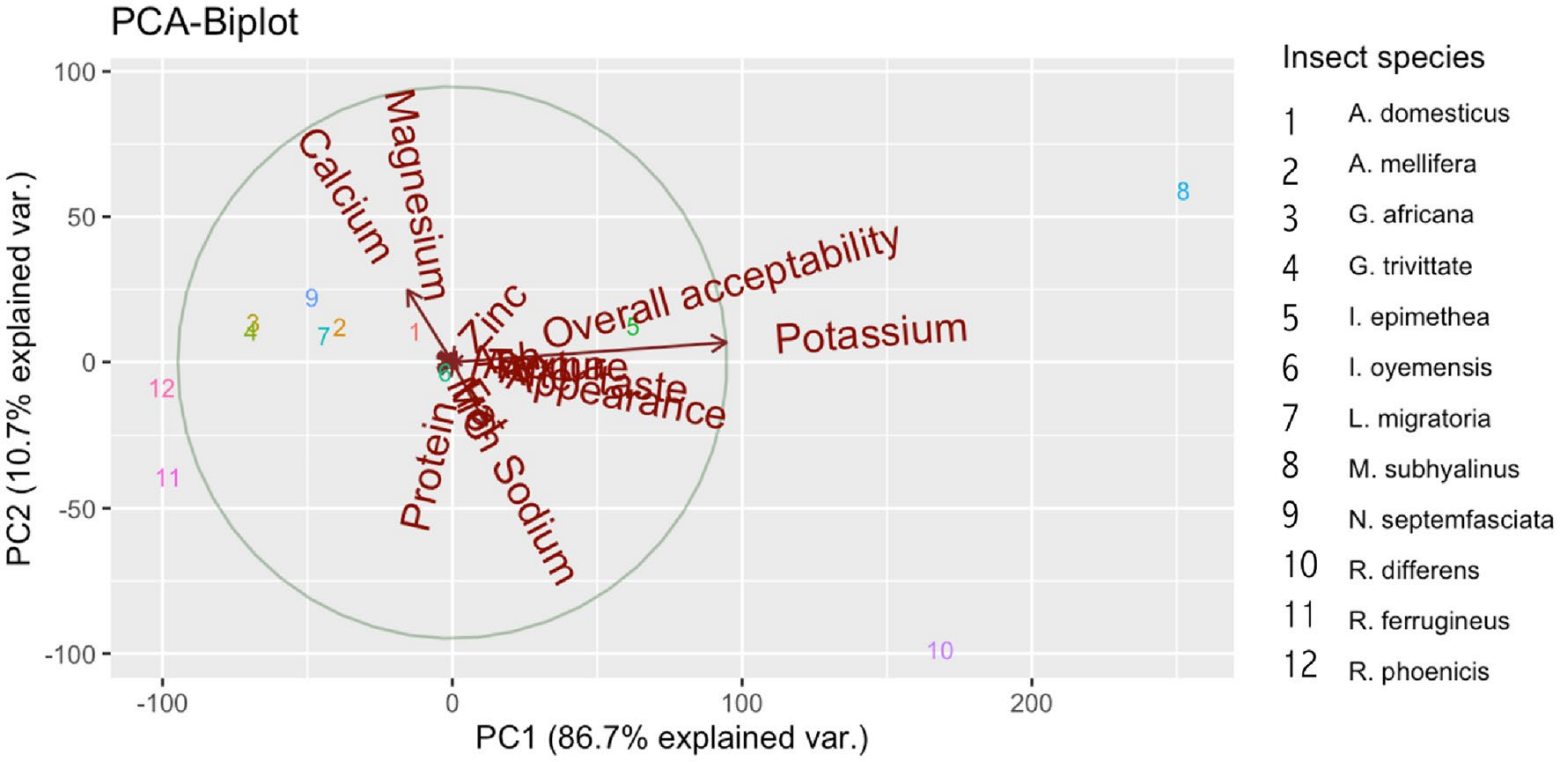
PCA-Biplot of nutrient composition and sensory attributes.

A scatter plot of matrices (SPLOM), histograms, and Pearson correlations between sensory attributes and macronutrient composition showed a strong positive correlation between fat content and texture, aftertaste, and overall acceptability, as depicted in Fig. 1. There was a strong correlation between appearance and aftertaste and overall acceptability. Overall acceptability correlated positively with aftertaste, texture, and aroma. Figure 2 showed a SPLOM, histograms, and Pearson correlations between sensory attributes and mineral profile. While a positive correlation was observed between iron and sodium content, a negative correlation was observed between calcium and the latter. In Fig. 3, the principal component analysis (PCA-Biplot) results indicated that the two axes accounted for up to 97.4% of the observed variability in the nutrient composition and sensory attributes of commonly consumed edible insects in the Eastern D. R. Congo. The first and second axes accounted for 86.7% and 10.7% of variability, respectively.

## Discussion

The nutritional composition of the edible insects reported in this study varies from one species to another, confirming the variability in nutritional composition of edible insects highlighted in the literature stipulating that the latter may vary according to species, insect life stage, type of insect feeding, habitat^23^, origin^34^, geographical distribution^38^, seasonal^38^ and environmental factors^39^ and preparation method^40^. In addition, nutritional composition varies between orders^41^ and even between species within the same order^34^. Moreover, the macronutrient composition of edible insects in this study is similar to the one reported by Rumpold & Schlüter^34^, who highlighted that the average protein content in edible insects is 40.69 g/100 g and varies between 6.25 and 71.10 g/100 g, 3.8 and 77.17 g/100 g for fat content, and ash content (1.35 and 12.85 g/100 g) on dry matter. The observed variation in nutritional composition of assessed edible insects are most probably linked to species^42^, feeding^43^, processing methods^40^, geographical sourcing area^38^, and measurement methods.

Specifically, the protein, fat, ash, and moisture content of *A. domesticus* observed in this study is lower than the protein (75.76 g/100 g) and moisture content (70 g/100 g) but higher than the fat (12.93 g/100 g), and ash content (4.54 g/100 g) reported by Bawa et al.^44^. Nevertheless, the macronutrient composition of *A. domesticus* is similar to the one reported by Montowska et al.^45^, and relatively comparable to that reported by Ayieko et al.^46^, and Oonincx et al.^5^. The macronutrient composition of *A. mellifera* reported in this study is similar to that reported by Ghosh et al.^47^, except the protein content, which is 35.3 g/100 g for larvae, 45.9 g/100 g (pupae) and 51 g/100 g (adults) on dry matter, and higher than the one reported by Agbidye et al.^48^.

Although less documented, the macronutrient composition of *G. trivittata* species found in this study corroborates that presented by Amouzou et al.^49^ in Togo, but with different protein contents. This difference would result from differences in agroecological conditions under which the species was harvested^38,43^. The macronutrient composition of *G. africana*, *I. oyemensis* and *N. septemfasciata* is in some cases superior to that of several conventional meats^23,50,51^, in addition to being tasty^25^, economic^11,18^, and environmentally friendly^52^.

On dry basis matter, the macronutrient composition of *I. epimethea* is superior to that of the same species purchased alive at local markets in Cameroon^53^, but lower than that degutted *I. epimethea* presented by Lautenschläger et al.^54^ in Angola. The protein content of *L. migratoria* in this study is lower than that reported in a study from Thailand, but it has a high fat content and similar ash content^55^. In this study, *M. subhyalinus* presented a macronutrient composition similar to that presented by Kinyuru et al.^41^ in Kenya with a protein content of 39.34 g/100 g and ash (7.78 g/100 g) but with a lower fat content (44.82 g/100 g).

Furthermore, the protein and lipid content of *R. phoenicis* in this study is superior to that reported by Mba et al.^56^ in Cameroon and Rumpold and Schlüter^34^, who reported contents varying between 10.3 and 35.6 g/100 g on dry weight basis. The protein (20.4 g/100 g) and ash content (3.5 g/100 g) of *R. ferrugineus*^57^ are lower than those reported in this study. However, they also reported lipid content (38.2 g/100 g) higher than those observed in this study. In this study, the protein content of *R. differens* was higher than that reported by Ssepuuya et al.^38^. On a dry matter basis; the average protein is similar to that observed in previous studies^58,59^. This study shows that consumption of 100 g of the studied edible insects can contribute to the daily protein requirement of 0.8–1.0 g/kg body weight^60^, and therefore, contribute to improving the low daily per capita protein consumption of 55–65 g/person/day in Sub-Saharan Africa^61^.

Similarly, the mineral profile, i.e. levels of potassium, sodium, magnesium, iron, calcium and zinc, varied significantly between edible insect species with potassium content ranging from 24 to 386.67 mg/100 g, sodium (152.27–257.82 mg/100 g), magnesium (32–64 mg/100 g), iron (5.30–16.13 mg/100 g), calcium (25.67–156.67 mg/100 g) and zinc (11–19. 67 mg/100 g), the mineral profile reported in this study is either similar or higher than those reported by Rumpold and Schlüter^34^, who reported potassium content ranging between 1.49 and 21800 mg/100 g, 20–2418 mg/100 g (sodium), 0.09–1910 mg/100 g (magnesium), 0.35–1562 mg/100 g (iron), 0.04–2010 mg/100 g (calcium), and 0.10–59 mg/100 g (zinc). The mineral profile of *A. domesticus* in this study is lower than or similar to that reported by Bawa et al.^44^ with 4.6 mg/100 g for iron, 21.63 mg/100 g (zinc), 183.89 mg/100 g (calcium), 398.84 mg/100 g (sodium) and 995.42 mg/100 g (potassium) on dry weight basis for fresh *A. domesticus*. On the other hand, the zinc and iron content observed in this study is higher than or comparable to that observed by Montowska et al.^45^, who reported a content of 12.8–21.8 mg/100 g and 4.06–5.99 mg/100 g for zinc and iron respectively.

Zinc, calcium, iron, magnesium, sodium and potassium content of *A. mellifera* observed in this study is similar, comparable or lower than that reported by Ghosh et al.^47^, depending on the mineral, who reported a zinc content of 11.6–14 mg/100 g, 84.9–222.9 mg/100 g (calcium), 13.3–37.7 mg/100 g (iron), 177–201.7 mg/100 g (magnesium), 59.4–75.6 mg/100 g (sodium) and 1585.4–2207.3 mg/100 g for potassium. Furthermore, the zinc and iron content in this study is comparable or higher than the zinc (12.8–21.8 mg/100 g) and iron (4.06–5.99 mg/100 g) content reported by Montowska et al.^45^. The mineral profile of *G. trivittata* in this study is superior, comparable or inferior to the potassium (1102.4 mg/100 g), sodium (44.80 mg/100 g), magnesium (33.43 mg/100 g), iron (1.65 mg/100 g), calcium (66.54 mg/100 g) and zinc (13.59 mg/100 g) content of *G. trivittata* collected in Togo^49^.

The iron, zinc and magnesium content of *M. subhylanus* observed are comparable to those reported in a previous study, which reported mineral contents of 6.2–10.3 mg/100 g (iron), 4.9–13.8 mg/100 g (zinc) and 39.8 mg/100 g (magnesium) for *M. subhylanus* and *Macrotermes* spp collected in Benin, and 8.8–9.8 mg/100 g (iron) and 12–12.9 mg/100 g (zinc) for *Odontotermes* spp and *Macrotermes* spp collected in South Africa, and 13.9 mg/100 g (iron), 12.9 mg/100 g (zinc) and 95 mg/100 g (magnesium) for *Odontotermes* spp collected in South East Asia^62^. Additionally, Omotoso and Adedire^63^ reported mineral contents ranging from 13.67 to 17 mg/kg (sodium), 372.5–457.5 mg/kg (potassium), 43.52–60.69 mg/kg (magnesium), 6–22.90 mg/kg (iron), 0.27–2.63 mg/kg (calcium) and 0.31–0.56 mg/kg (zinc) which are for some mineral inferior and comparable to other mineral in comparison to the mineral profile of *R. phoenicis* observed in this study.

As for *R. differens*, the potassium, sodium, magnesium, iron, calcium and zinc content observed in this study is comparable to the potassium (259.7–370.6 mg/100 g), sodium (229.7–358.7 mg/100 g), magnesium (33.1–33.9 mg/100 g), iron (13–16.6 mg/100 g), calcium (24.5–27.4 mg/100 g) and zinc (12.4–17.3 mg/100 g) content reported by Kinyuru et al.^59^. Low levels of calcium have been reported in *R. differens* and other grass-hoppers as well^64^. Though the low calcium levels can be attributed to insects lacking a mineralized skeleton, animal sources with a mineralized skeleton, such as pork and chicken meat, have a lower calcium content. However, Fombong and collaborators^58^ reported higher amounts of calcium, ranging from 977 to 1124 mg/100 g of dried *R. differens*. This considerable variation within the same species can result from the diet/feed to which the *R. differens* are exposed in the different sourcing geographical areas and swarming seasons. The sodium content is much lower than that observed in other edible grasshoppers and other edible insects^34^. Though the magnesium content values are similar to those observed by Kinyuru et al.^59^, they are lower than those observed by Fombong et al.^58^ in the same species. Compared to pork and chicken meat, *R. differens* is a better source of dietary macro- and micro-mineral elements. Hence, like other animal foods, *R. differens* can potentially contribute to alleviating the effects of zinc and iron micro-mineral deficiencies, which are among the most important micro-nutrients of public health concern, especially in Africa^65^.

Although poorly documented in terms of mineral profile, the mineral profile of *G. africana*, *I. epimethea*, *I. oyemensis*, *L. migratoria*, *N. septemfasciata* and *R. ferrugineus* observed in this study meets the mineral requirements for adults and children, especially for zinc and iron^66^. It has been observed that locusts have twenty-seven minerals^67^. The mineral content in 100 g of *L. migratoria* on dry matter varies between 8 and 20 mg^23^. In 2018, a study reported that *L. migratoria* contains an equal amount of zinc and a higher ratio of iron measured in mg/100 g dry matter and compared to poultry, beef and pork^68^.

In this study, acceptance of edible insects varied from one species to another, confirming the that social and cultural aspects are among the most important elements in their acceptance^69^. The development of delicious and healthy foods containing insects and the adoption of strategies would be an asset for the acceptance of edible insects in countries where their acceptance is still a challenge and are often considered disgusting, although their taste is proven to be mild and easy to accept^10^. Although edible insects are gaining momentum, familiarity seems to be the main driving force, allowing most people to react positively to all edible species in terms of willingness to eat them. Thus, appreciation is linked to availability^70^, ethnicity/culture^71^, palatability^72^, and seasonality^41^. In addition, indigenous knowledge and processing can also influence the appreciation of edible insects^73^.

Findings in this study showed that *M. subhyalinus* had the highest sensory score, followed by *R. differens*, *N. septemfasciata*, *R. phoenicis*, *L. migratoria*, *G. africana*, *I. epimethea*, *A. mellifera*, *R. ferrugineus*, *I. oyemensis*, *G. trivittata* and *A. domesticus* had the lowest sensory score based on overall acceptability, confirming the previous studies of Ishara et al.^24,25^, who found by a survey that *R. differens*, *M. subhyalinus*, *R. phoenicis* and *L. migratoria* were among the most appreciated edible insects in Eastern Democratic Republic of Congo. Furthermore, previous studies in Portugal^74^, United States of America^75,76^, Italy^77^ and Belgium^78^ reported that *A. domesticus* is overall-liked.

Other studies conducted in Nigeria^79^ and the USA^75^ demonstrated that termites and *L. migratoria* were overall liked. Moreover, several studies have shown that education would be crucial for a positive attitude towards edible insects among consumers^80^. A study in the Netherlands showed that people who had previously eaten insects had significantly more positive attitudes towards entomophagy than those who had never eaten them and were more likely to eat them again^81^.

The relationship between nutrient composition and sensory acceptance is a dynamic and intricate interplay that significantly influences our food choices, dietary habits, and overall health^82^. It is a complex interplay that involves not only the nutritional value of a food but also how it appeals to our senses, including taste, smell, texture, and appearance^83^. This connection is essential for understanding how the nutritional content of foods impacts their appeal and acceptability among consumers^78^. Taste is a fundamental factor in sensory acceptance. It is closely tied to nutrient composition, although research shows that individuals have variable taste preferences, which can be influenced by genetics and culture^82^.

On the other hand, the aroma of a food, closely linked to its flavour, is another critical aspect of sensory acceptance. Aromas are mainly produced by volatile compounds present in foods, and these compounds are influenced by nutrient composition. Nutrient-rich foods often have more complex and appealing aromas^68^. The texture of a food is essential for sensory acceptance and is determined by nutrient composition^83^. Factors such as fat content, water content and the presence of different textures, such as crunchiness or creaminess, have a significant impact on a food's sensory appeal. Research has shown that fat content influences creaminess and mouthfeel, contributing to sensory satisfaction^84^. Appearance (visual appeal) is an essential component of sensory acceptance. Nutrient composition is vital in food appearance, including color, shape and overall presentation^83^. Attractive colors are often indicative of nutrient richness^85^. A visually appealing food is more likely to be accepted, even before the first bite^85^.

## Conclusion and recommendations

The edible insects studied here are highly nutritious, showing their potential as a good source of nutrients with impressive appreciation, underlining their importance in tackling the issues of food insecurity. Although the edible insects studied are nutritionally rich with good sensory scores, these qualities are largely subjected to insect species. In order to fully assess the contribution of the studied edible insects to food and feed security in food-insecure countries, it is necessary to study their protein quality as a source of essential amino acids and investigate their fatty acids profile, safety, nutrient digestibility and bioavailability as well as the influence of processing on their nutritional quality in addition to encouraging mass rearing to respond to their high existing demand.

## Material and methods

### Ethics approval

All experimental protocols, as well as methods, were approved and carried out as per relevant guidelines and regulations from the Interdisciplinary Centre for Ethical Research (CIRE) established by the Université Evangélique en Afrique, Bukavu, D.R. Congo, with reference (UEA/SGAC/KM 132/2016).

### Sample collection

**Figure 4:**
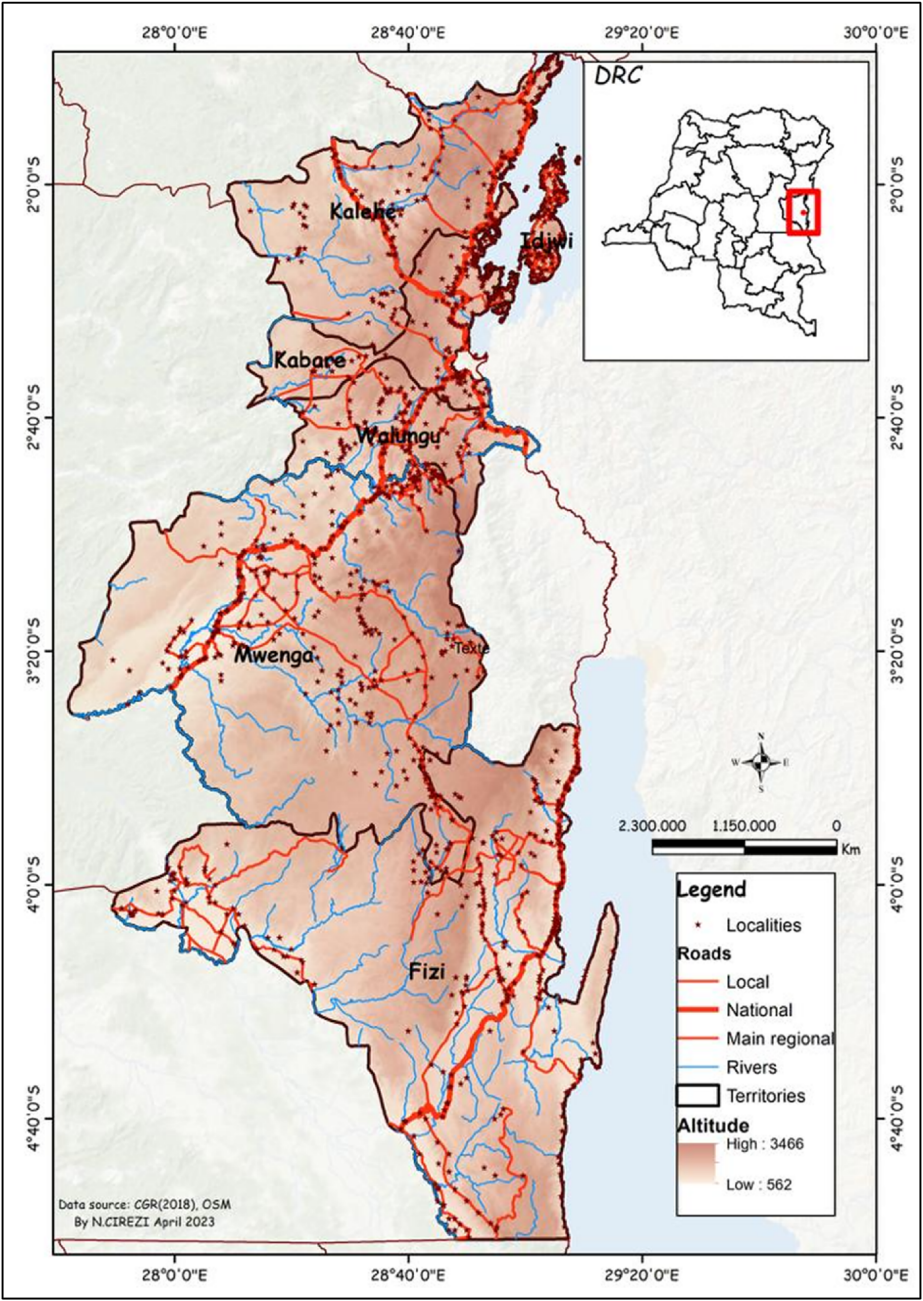
Map showing the Democratic Republic of the Congo, the South-Kivu Province, and the study area.

About 5 kg of each commonly edible insect namely *Acheta domesticus*, *Apis mellifera* larvae, *Gnathocera trivittata*, *Gryllotalpa africana*, *Imbrasia epimethea*, *Imbrasia oyemensis, Locusta migratoria, Macrotermes subhylanus, Nomadacris septemfasciata*, *Rhyncophorus phoenicis, Ruspolia differens* and *Rhynchophorus ferrugineus* were collected from six geographical areas namely Fizi, Idjwi, Kabare, Kalehe, Mwenga and Walungu, in Eastern Democratic Republic of Congo as mapped in Fig. 4. The territories were purposely selected for their familiarity with entomophagy practices and unique agroecological conditions, thus influencing edible insects’ potential as food and feed.

### Sample preparation

**Figure 5:**
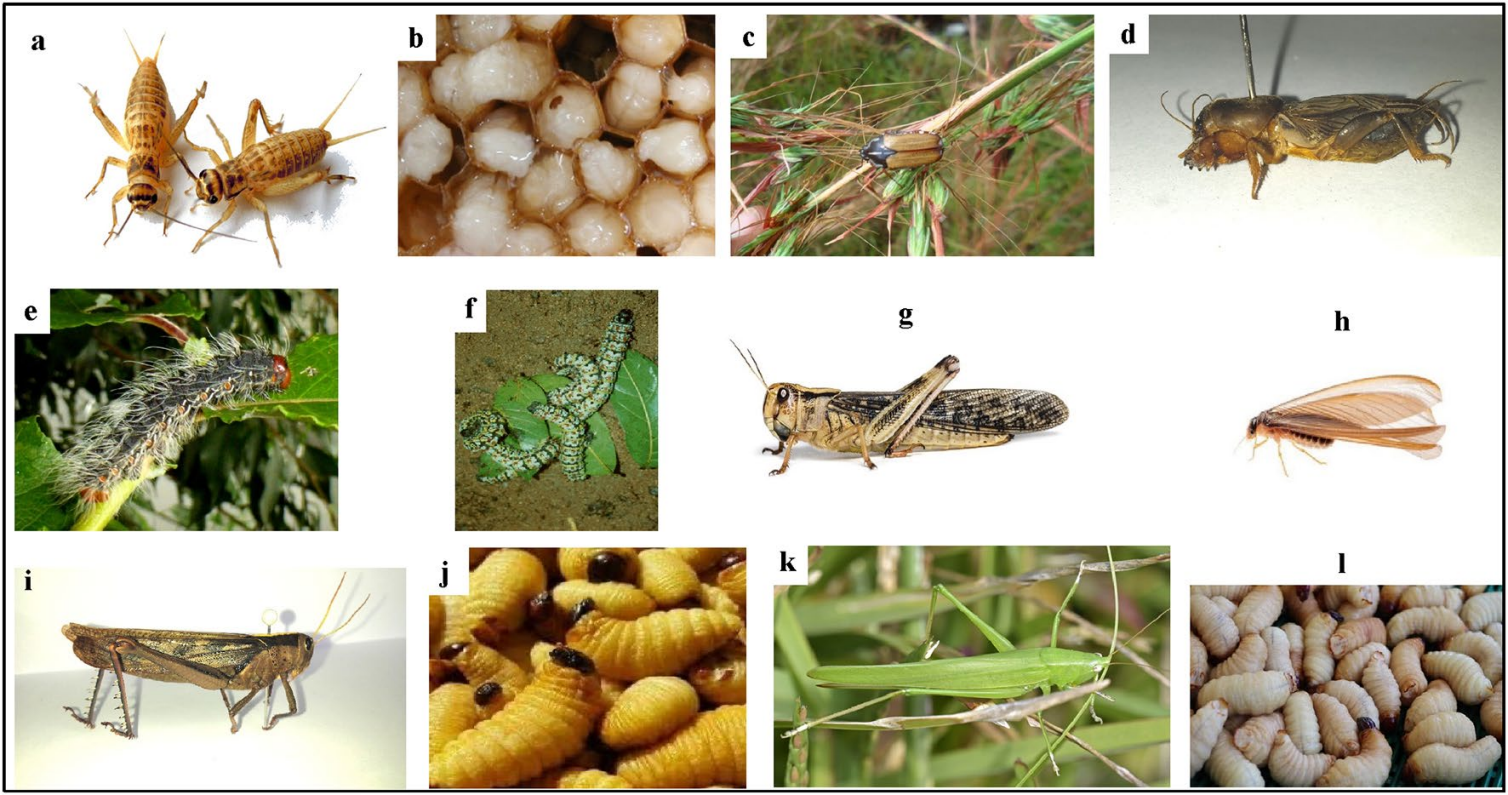
(**a**) *Acheta domesticus* (House cricket); (**b**) *Apis mellifera* larvae (Honey bee); (**C**) *Gnathocera trivittata* (Nsike); (**d**) *Gryllotalpa africana* (Mole cricket); (**e**) *Imbrasia epimethea* (Caterpillar); (**f**) *Imbrasia oyemensis (*Caterpillar); (**g**) *Locusta migratoria* (Migratory locust); (**h**) *Macrotermes subhylanus* (Termite); (**i**) *Nomadacris septemfasciata* (Red locust); (**j**) *Rhyncophorus phoenicis* larvae; (**k**) *Ruspolia differens* (Grasshopper); (**l**) *Rhynchophorus ferrugineus* larvae known as Red palm weevil (Ishara et al.^25^. Figure modified and reproduced with permission from Springer Nature http://creativecommons.org/licenses/by/4.0/).

Edible insect samples (Fig. 5) from each geographical sourcing area were harvested using traditional methods as described by Ishara et al.^24,25^, then packed in zipping polyethylene bags and delivered to Université Evangelique en Afrique on flaked ice in a cool box before being washed clean and drained. About half of the samples were frozen at − 20 °C shortly until further analyses, and the other half was directly used for sensory assessment purposes.

### Macronutrient composition

Macronutrient composition was determined in accordance with the Association of Official Analytical Chemists^86^. While moisture and ash were determined by the hot-air circulating oven (105 °C) and through incineration in a muffle furnace (600 °C) respectively, crude fat content was determined by solvent extraction method using SoxtecTM2055. Crude protein was determined by the Kjeldahl method and its content was obtained by multiplying the corresponding total nitrogen content by a factor of 5.33^87^. All determinations were carried in triplicate and expressed as mean ± standard error.

### Mineral composition

Potassium, Sodium, Magnesium, Iron, Calcium and Zinc were determined in accordance with Association of Official Analytical Chemists^86^. Samples were ashed and the residue dissolved with HCl and filtered using a Whatman filter paper. The mineral content was determined using AA-7000 Atomic Absorption Spectrophotometer (AAS-Shimadzu Corporation, Japan). The absorbance of sample and standard solutions was determined.

### Sensory evaluation

Insects were cooked using the methods described by Ishara et al.^24,25^ as shown in Table 4. *A. domesticus* and *N. septemfasciata* were deep-fried for 7 min, *A. mellifera*, *I. oyemensis, I. epimethea, R. phoenicis* and *R. ferrugineus* were boiled, roasted and deep fried for 10 min, *G. trivittata* and *G. africana* were deep-fried for 10 min. Finally, *M. subhyalinus* and *R. differens* were fried for 5 min.

**Table 4:** Cooking time and ingredients used.

Insects	Water (cl)	Salt (g)	Oil (cl)	Cooking time (min)
*Acheta domesticus*	0	5	5	7
*Apis mellifera*	10	5	0	10
*Gnathocera trivittata*	0	5	5	10
*Grillotalpa africana*	0	5	5	10
*Imbrasia epimethea*	0	2	2	10
*Imbrasia oyemensis*	0	2	2	10
*Locusta migratoria*	0	5	5	7
*Macrotermes subhyalinus*	0	5	2	5
*Nomadacris septemfasciata*	0	5	5	7
*Rhynchophorus*				
*phoenicis*	0	4	2	10
*Ruspolia differens*	0	5	2	5
*Rynchophorus ferrugineus*	0	2	2	10

Sensory evaluation of the cooked edible insects was carried out at room temperature shortly after cooking by forty panellists from the Université Evangélique en Afrique (UEA). Each cooked edible insect’s sample was placed on a small plastic plate and labelled with a random three-digit number. Between sample tests, panellists used neutral non-carbonated mineral water to rinse the mouth. The samples were evaluated in relation to appearance, aroma, taste, texture and overall score was carried out with an intensity-based questionnaire using a 7-point hedonic scale (1 = dislike extremely, 2 = dislike moderately, 3 = dislike slightly, 4 = neither like or dislike, 5 = like slightly, 6 = like moderately and 7 = like extremely) according to Ihekoronye and Ngoddy^88^.

### Statistical analysis

Data collected in triplicates were encoded in Microsoft Excel for Mac (Version 16.74). R-Studio Version 4.2.0 and Statistix Version 10 Software were used for statistical analysis including correlation as well as principal component analysis (PCA-Biplot), and data were presented as mean ± standard error. Analysis of variance (ANOVA) was used to compare the nutritional composition and sensory attributes of wild harvested edible insects consumed in the Eastern D.R. Congo. Means were separated using Tukey's test at a significance level of 0.05.

## Data Availability

The data supporting the findings reported herein are available on reasonable request from the corresponding author.

## References

[1] Desa UN (2019). World population prospects 2019: Highlights. N. Y. U. N. Dep. Econ. Soc. Aff..

[2] Tabari H (2020). Climate change impact on flood and extreme precipitation increases with water availability. Sci. Rep..

[3] Ishara J, Ogunyiola A, Matendo R, Kiyala JCK, Karume K, Kiyala JCK, Chivasa N (2024). Climate Change and Its Implications on Food Security in the Great Lakes Region. Climate change and socio-political violence in Sub-Saharan Africa in the anthropocene: perspectives from peace ecology and sustainable development.

[4] Godfray HCJ (2010). Food security: The challenge of feeding 9 billion people. Science.

[5] Van Huis A, Oonincx DGAB (2017). The environmental sustainability of insects as food and feed. A review. Agron. Sustain. Dev..

[6] Gerten D (2020). Feeding ten billion people is possible within four terrestrial planetary boundaries. Nat. Sustain..

[7] Omuse ER (2024). The global atlas of edible insects: analysis of diversity and commonality contributing to food systems and sustainability. Sci. Rep..

[8] Nowakowski AC, Miller AC, Miller ME, Xiao H, Wu X (2022). Potential health benefits of edible insects. Crit. Rev. Food Sci. Nutr..

[9] Wendin KME, Nyberg ME (2021). Factors influencing consumer perception and acceptability of insect-based foods. Curr. Opin. food Sci..

[10] Mishyna M, Chen J, Benjamin O (2020). Sensory attributes of edible insects and insect-based foods–Future outlooks for enhancing consumer appeal. Trends Food Sci. Technol..

[11] Dobermann D, Swift JA, Field LM (2017). Opportunities and hurdles of edible insects for food and feed. Nutr. Bull..

[12] Baiano A (2020). Edible insects: An overview on nutritional characteristics, safety, farming, production technologies, regulatory framework, and socio-economic and ethical implications. Trends Food Sci. Technol..

[13] Lumanlan JC, Williams M, Jayasena V (2022). Edible insects: Environmentally friendly sustainable future food source. Int. J. Food Sci. Technol..

[14] Panassiti B (2023). Insects benefit from agri-environmental schemes aiming at grassland extensification. Agric. Ecosyst. Environ..

[15] Poma G (2017). Evaluation of hazardous chemicals in edible insects and insect-based food intended for human consumption. Food Chem. Toxicol..

[16] Hanboonsong Y, Jamjanya T, Durst PB (2013). Six-Legged Livestock: Edible Insect Farming, Collection and Marketing in Thailand.

[17] Hope RA, Frost PGH, Gardiner A, Ghazoul J (2009). Experimental analysis of adoption of domestic mopane worm farming technology in Zimbabwe. Dev. South. Afr..

[18] Tanga CM (2021). Edible insect farming as an emerging and profitable enterprise in East Africa. Curr. Opin. insect Sci..

[19] Govorushko S (2019). Global status of insects as food and feed source: A review. Trends Food Sci. Technol..

[20] Jongema, Y. List of edible insects of the world, Table, Lab. Entomol., Wageningen Univ., Neth. https://www.wur.nl/en/Research-Results/Chair-groups/Plant-Sciences/Laboratory-of-Entomology/Edible-insects/Worldwide-species-list.htm. (2019).

[21] Tang C (2019). Edible insects as a food source: a review. Food Prod. Process. Nutr..

[22] Premalatha M, Abbasi T, Abbasi T, Abbasi SA (2011). Energy-efficient food production to reduce global warming and ecodegradation: The use of edible insects. Renew. Sustain. energy Rev..

[23] Van Huis A (2013). Potential of insects as food and feed in assuring food security. Annu. Rev. Entomol..

[24] Ishara J (2022). Inventory reveals wide biodiversity of edible insects in the Eastern Democratic Republic of Congo. Sci. Rep..

[25] Ishara J (2023). Edible insect biodiversity and anthropo-entomophagy practices in Kalehe and Idjwi territories. DR Congo. J. Ethnobiol. Ethnomed..

[26] Bomolo O (2017). Ecological diversity of edible insects and their potential contribution to household food security in Haut-Katanga Province, Democratic Republic of Congo. Afr. J. Ecol..

[27] Payne CLR, Mato B, Fruth B (2016). Entomophagy in the area surrounding LuiKotale, Salonga National Park, Democratic Republic of the Congo. Afr. Study Monogr..

[28] Muvatsi P, Kahindo J-M, Snook LK (2018). Can the production of wild forest foods be sustained in timber concessions? Logging and the availability of edible caterpillars hosted by sapelli (*Entandrophragma cylindricum*) and tali (*Erythrophleum suaveolens*) trees in the Democratic Republic of Congo. For. Ecol. Manage..

[29] Cuni-Sanchez A (2019). Social perceptions of forest ecosystem services in the Democratic Republic of Congo. Hum. Ecol..

[30] Ojha S, Bekhit AE-D, Grune T, Schlüter OK (2021). Bioavailability of nutrients from edible insects. Curr. Opin. Food Sci..

[31] Ishara J (2022). Nutraceutical potential of mushroom bioactive metabolites and their food functionality. J. Food Biochem..

[32] Dumrongwongsiri O (2022). Zinc and iron adequacy and relative importance of zinc/iron storage and intakes among breastfed infants. Matern. Child Nutr..

[33] Anaduaka EG, Uchendu NO, Osuji DO, Ene LN, Amoke OP (2021). Nutritional compositions of two edible insects: *Oryctes rhinoceros larva* and *Zonocerus variegatus*. Heliyon.

[34] Rumpold BA, Schlüter OK (2013). Nutritional composition and safety aspects of edible insects. Mol. Nutr. Food Res..

[35] Borges MM, da Costa DV, Trombete FM, Câmara AKFI (2022). Edible insects as a sustainable alternative to food products: an insight into quality aspects of reformulated bakery and meat products. Curr. Opin. Food Sci..

[36] Belluco S (2013). Edible insects in a food safety and nutritional perspective: a critical review. Compr. Rev. food Sci. food Saf..

[37] Hawkey KJ, Lopez-Viso C, Brameld JM, Parr T, Salter AM (2021). Insects: a potential source of protein and other nutrients for feed and food. Annu. Rev. Anim. Biosci..

[38] Ssepuuya G, Smets R, Nakimbugwe D, Van Der Borght M, Claes J (2019). Nutrient composition of the long-horned grasshopper *Ruspolia differens* Serville: Effect of swarming season and sourcing geographical area. Food Chem..

[39] Leonard A (2022). Host plant-based artificial diets enhance development, survival and fecundity of the edible long-horned grasshopper *Ruspolia differens* (orthoptera: Tettigoniidae). J. Insect Sci..

[40] Mishyna M, Keppler JK, Chen J (2021). Techno-functional properties of edible insect proteins and effects of processing. Curr. Opin. Colloid Interface Sci..

[41] Kinyuru JN (2013). Nutrient composition of four species of winged termites consumed in western Kenya. J. Food Compos. Anal..

[42] Chen X, Feng Y, Chen Z (2009). Common edible insects and their utilization in China. Entomol. Res..

[43] Ramos-Elorduy J, González EA, Hernández AR, Pino JM (2002). Use of *Tenebrio molitor* (Coleoptera: Tenebrionidae) to recycle organic wastes and as feed for broiler chickens. J. Econ. Entomol..

[44] Bawa M, Songsermpong S, Kaewtapee C, Chanput W (2020). Nutritional, sensory, and texture quality of bread and cookie enriched with house cricket (*Acheta domesticus*) powder. J. Food Process. Preserv..

[45] Montowska M, Kowalczewski PŁ, Rybicka I, Fornal E (2019). Nutritional value, protein and peptide composition of edible cricket powders. Food Chem..

[46] Ayieko MA, Kinyuru JN, Ndong’a MF, Kenji GM (2012). Nutritional value and consumption of black ants (*Carebara vidua Smith*) from the Lake Victoria region in Kenya. Adv. J. Food Sci. Technol..

[47] Ghosh S, Jung C, Meyer-Rochow VB (2016). Nutritional value and chemical composition of larvae, pupae, and adults of worker honey bee, *Apis mellifera ligustica* as a sustainable food source. J. Asia. Pac. Entomol..

[48] Agbidye FS, Ofuya TI, Akindele SO (2009). Marketability and nutritional qualities of some edible forest insects in Benue State, Nigeria. Pak. J. Nutr..

[49] Amouzou K, Tete-Benissan A, Badanaro F (2021). Nutritional potential of two insect species consumed in Togo: *Gnathocera trivittata* (Swederus, 1787) and *Gnathocera impressa* (Olivier, 1789). Eur. J. Nutr. Food Saf..

[50] Kinyuru JN, Halloran A, Flore R, Vantomme P, Roos N (2018). The Role of edible insects in diets and nutrition in east Africa. Edible Insects in Sustainable Food Systems.

[51] Payne CLR, Scarborough P, Rayner M, Nonaka K (2016). A systematic review of nutrient composition data available for twelve commercially available edible insects, and comparison with reference values. Trends Food Sci. Technol..

[52] Bang A, Courchamp F (2021). Industrial rearing of edible insects could be a major source of new biological invasions. Ecol. Lett..

[53] Mba ARF, Kansci G, Viau M, Rougerie R, Genot C (2019). Edible caterpillars of *Imbrasia truncata* and *Imbrasia epimethea* contain lipids and proteins of high potential for nutrition. J. Food Compos. Anal..

[54] Lautenschläger T, Neinhuis C, Kikongo E, Henle T, Förster A (2017). Impact of different preparations on the nutritional value of the edible caterpillar *Imbrasia epimethea* from northern Angola. Eur. Food Res. Technol..

[55] Brogan EN, Park Y-L, Matak KE, Jaczynski J (2021). Characterization of protein in cricket (*Acheta domesticus*), locust (*Locusta migratoria*), and silk worm pupae (*Bombyx mori*) insect powders. LWT.

[56] Mba ARF (2017). Lipid and amino acid profiles support the potential of *Rhynchophorus phoenicis* larvae for human nutrition. J. Food Compos. Anal..

[57] Chinarak K (2021). Insights into the effects of dietary supplements on the nutritional composition and growth performance of sago palm weevil (*Rhynchophorus ferrugineus*) larvae. Food Chem..

[58] Fombong FT, Van Der Borght M, Vanden Broeck J (2017). Influence of freeze-drying and oven-drying post blanching on the nutrient composition of the edible insect *Ruspolia differens*. Insects.

[59] Kinyuru JN, Kenji GM, Muhoho SN, Ayieko M (2010). Nutritional potential of longhorn grasshopper (*Ruspolia differens*) consumed in Siaya district Kenya. J. Agric. Sci. Technol..

[60] Whyte G, Loosemore M, Williams C (2015). ABC of Sports and Exercise Medicine.

[61] Oecd F (2016). Agriculture in sub-Saharan Africa: Prospects and challenges for the next decade. OECD-FAO Agric. Outlook.

[62] Verspoor RL (2020). Mineral analysis reveals extreme manganese concentrations in wild harvested and commercially available edible termites. Sci. Rep..

[63] Omotoso OT, Adedire CO (2007). Nutrient composition, mineral content and the solubility of the proteins of palm weevil, *Rhynchophorus phoenicis* f. (Coleoptera: Curculionidae). J. Zhejiang Univ. Sci. B.

[64] Paul A (2016). Grasshoppers as a food source? A review. Biotechnol. Agron. Société Environ..

[65] Harika R (2017). Micronutrient status and dietary intake of iron, vitamin A, iodine, folate and zinc in women of reproductive age and pregnant women in Ethiopia, Kenya, Nigeria and South Africa: A systematic review of data from 2005 to 2015. Nutrients.

[66] FAO, WHO (2004). Evaluation of certain food additives and contaminants: sixty-first report of the Joint FAO/WHO Expert Committee on Food Additives.

[67] Yin W, Liu J, Liu H, Lv B, Mikkola H (2017). Nutritional value, food ingredients, chemical and species composition of edible insects in China. Future Foods.

[68] Mwangi MN (2018). Insects as sources of iron and zinc in human nutrition. Nutr. Res. Rev..

[69] Elhassan M, Wendin K, Olsson V, Langton M (2019). Quality aspects of insects as food—nutritional, sensory, and related concepts. Foods.

[70] Van Huis A (2013). Edible Insects: Future Prospects for Food and Feed Security.

[71] Riggi LG, Veronesi M, Goergen G (2016). Observations of entomophagy across Benin—practices and potentials. Food Secur..

[72] Halloran A, Vantomme P, Hanboonsong Y, Ekesi S (2015). Regulating edible insects: The challenge of addressing food security, nature conservation, and the erosion of traditional food culture. Food Secur..

[73] Obopile M, Seeletso TG (2013). Eat or not eat: An analysis of the status of entomophagy in Botswana. Food Secur..

[74] Cunha LM, Ribeiro JC, Sogari G, Mora C, Menozzi D (2019). Sensory and consumer perspectives on edible insects. Edible Insects in the Food Sector: Methods, Current Applications and Perspectives.

[75] Tao J, Davidov-Pardo G, Burns-Whitmore B, Cullen EM, Li YO (2017). Effects of edible insect ingredients on the physicochemical and sensory properties of extruded rice products. J. Insects Food Feed.

[76] Zhong A (2017). Product development considerations for a nutrient rich bar using cricket (Acheta domesticus) protein.

[77] Sogari G, Menozzi D, Mora C (2018). Sensory-liking expectations and perceptions of processed and unprocessed insect products. Int. J. Food Syst. Dyn..

[78] Megido CR (2014). Edible insects acceptance by *B elgian* consumers: promising attitude for entomophagy development. J. Sens. Stud..

[79] Ogunlakin GO, Oni VT, Olaniyan SA (2018). Quality evaluation of biscuit fortified with edible termite (*Macrotermes nigeriensis*). Asian J. Biotechnol. Bioresour. Technol..

[80] Looy H, Wood JR (2006). Attitudes toward invertebrates: Are educational" bug banquets" effective?. J. Environ. Educ..

[81] Smith ABT, Paucar A (2000). Taxonomic review of *Platycoelia lutescens* (Scarabaeidae: Rutelinae: Anoplognathini) and a description of its use as food by the people of the Ecuadorian highlands. Ann. Entomol. Soc. Am..

[82] Reed DR, Tanaka T, McDaniel AH (2006). Diverse tastes: Genetics of sweet and bitter perception. Physiol. Behav..

[83] Pan X, Bi S, Lao F, Wu J (2023). Factors affecting aroma compounds in orange juice and their sensory perception: A review. Food Res. Int..

[84] Lucey JA (2004). Cultured dairy products: An overview of their gelation and texture properties. Int. J. Dairy Technol..

[85] Wolfe KL, Liu RH (2003). Apple peels as a value-added food ingredient. J. Agric. Food Chem..

[86] AOAC, B. A. M. Association of official analytical chemists. *Off. methods Anal.* **12**, (2000).

[87] Boulos S, Tännler A, Nyström L (2020). Nitrogen-to-protein conversion factors for edible insects on the Swiss market: *T. molitor*, *A. domesticus*, and *L. migratoria*. Front. Nutr..

[88] Ihekoronye AI, Ngoddy PO (1985). Integrated Food Science and Technology for the Tropics.

